# Evaluation of a Web-based tailored intervention (*TAVIE en santé*) to support people living with HIV in the adoption of health promoting behaviours: an online randomized controlled trial protocol

**DOI:** 10.1186/s12889-015-2310-4

**Published:** 2015-10-12

**Authors:** José Côté, Sylvie Cossette, Pilar Ramirez-Garcia, Alexandra De Pokomandy, Catherine Worthington, Marie-Pierre Gagnon, Patricia Auger, François Boudreau, Joyal Miranda, Yann-Gaël Guéhéneuc, Cécile Tremblay

**Affiliations:** Research Centre of the Centre Hospitalier de l’Université de Montréal, 900 Saint Denis Street, Montreal, H2X 0A9 QC Canada; Research Chair in Innovative Nursing Practices, 900 Saint Denis Street, Montreal, H2X 0A9 QC Canada; Faculty of Nursing, Université de Montréal, 2375, chemin de la Côte-Ste-Catherine, Montréal, H3T 1A8 QC Canada; Research Center of the Montreal Heart Institute, 5000, Bélanger Street, Montréal, H1T 1C8 QC Canada; Faculty of Medecine, McGill University, 3655 Sir William Osler, Montreal, H3G 1Y6 QC Canada; Faculty of Human and Social Development, University of Victoria, 3800 Finnerty Road, Victoria, V8P 5C2 BC Canada; Research Centre of the Centre Hospitalier Universitaire de Québec, 2705, boulevard Laurier, Québec, G1V 4G2 QC Canada; Faculty of Nursing Sciences, Université Laval, 1050, avenue de la Médecine Local 3645, Québec, G1V 0A6 QC Canada; Faculty of Nursing, Université du Québec à Trois-Rivièves, 3351, boul. des Forges, CP 500, Trois-Rivières, G9A 5H7 QC Canada; Ryerson University, 350 Victoria Street, Toronto, M5B 2K3 ON Canada; Canada Research Chair on Software Patterns and Patterns of Software, 2500, chemin de Polytechnique, Montréal, H3T 1J4 QC Canada; Department of Computer Engineering, Polytechnique Montréal, 2500, chemin de Polytechnique, Montréal, H3T 1J4 QC Canada; Quebec Public Health Laboratory, Sainte-Marie Rd, Sainte-Anne-de-Bellevue, H9X 3R5 QC Canada; Faculty of Medecine, Université de Montréal, 2900, boulevard Édouard-Montpetit, Montréal, H3T 1J4 QC Canada

**Keywords:** Online randomized control trial, Web-based tailored intervention, People living with HIV, Smoking cessation, Physical activity, Healthy eating, Intention, Attitude, Perceived control, Theory of planned behaviour

## Abstract

**Background:**

Long-term use of antiretroviral therapy, normal aging, and presence of certain risk factors are associated with metabolic disorders that predispose persons living with HIV to diabetes and cardiovascular diseases. The emergence and progression of these disorders can be prevented by adopting healthy behaviours. Based on the theory of planned behaviour, the Web-based tailored intervention *TAVIE en santé* was developed. The aim of this study is to evaluate the effectiveness of *TAVIE en santé* in order to support people living with HIV in the adoption of health promoting behaviours.

**Methods/Design:**

An online randomized controlled trial with parallel-groups will be conducted across Canada. To participate in this study, people living with HIV must be: ≥ 18 years, able to read/understand French or English, have access to the Internet. A convenience sample of 750 participants will be randomly assigned either to an experimental group (*TAVIE en santé*, *n* = 375) or to a control group (websites, *n* = 375) (1:1 allocation ratio). The *TAVIE en santé* intervention is composed of seven interactive computer sessions, lasting between 5 and 10 min. The sessions, hosted by a virtual nurse, aim to develop and strengthen skills required for behaviour change. The control group will receive a validated list of five predetermined conventional health-related Websites. The adoption of health behaviour (smoking cessation or physical activity or healthy eating) is the principal outcome. Cognitions (intention, attitude, perceived behavioral control) are the secondary outcomes. Health indicators will also be assessed. All outcomes will be measured with a self-administered online questionnaire and collected three times: at baseline, 3 and 6 months after. The principal analyses will focus on differences between the two trial groups using Intention-to-Treat analysis.

**Discussion:**

This study will yield new results about the efficacy of Web-based tailored health behaviours change interventions in the context of chronic disease. The *TAVIE en santé* intervention could constitute an accessible complementary service in support of existing specialized services to support people living with HIV adopt health behaviors.

**Trial registration:**

NCT02378766, assigned on March 3th 2015.

## Background

The advent of powerful antiretroviral therapy (ART) has contributed to a substantial reduction in morbidity and mortality among persons living with HIV (PLHIV). Formerly considered a terminal illness, HIV infection is now considered as a chronic disease [1–4] and new HIV-associated complications are increasingly emerging [3]. For instance, the occurence of metabolic disorders, including glucose metabolism dysfunction, dyslipidemia and cardiovascular diseases have been found to be associated with the long-term use of ART, the normal aging process, and the presence of certain risk factors [5–9]. Notably, compared to the general population, smoking is more prevalent [10–14] and physical activity levels are lower among PLHIV [15, 16]. Also, PLHIV are likely to exceed the guidelines for recommended saturated fat [17, 18] and cholesterol dietary intake [19]. Thus, to prevent the emergence and progression of comorbid problems, such as diabetes and cardiovascular diseases, it is essential to intervene actively on modifiable risk factors in order to help PLHIV adopt health promoting behaviours [20].

In the domain of health behaviour change interventions, the deployment and utilisation of information and communications technologies (ICT) as a way to deliver the interventions appear to be promising and has the potential to promote healthy behaviours [21, 22]. In their meta-analysis of Web-delivered tailored health behaviour change interventions (*n* = 40 randomized controlled trials), Lustria and colleagues (2013) reported that tailored, Web-based interventions had a significantly greater effect on healthy behaviours, such as smoking cessation, physical activity, healthy eating, than the comparison/control conditions (e.g., a non-tailored or waitlist control condition) [21]. However, web-based tailored interventions have been most successful when targeting general populations (*n* = 26, *d* = 0.181, 95 % confidence interval (CI) 0.148–0.214) rather than chonically ill patients (*n* = 6, *d* = 0.140, 95 % CI 0.037–0.244). Until recently, preventing comorbidities among PLHIV has involved more traditional interventions addressing mostly smoking cessation [13, 14], to promote and/or increase physical activity [23, 24] and to improve diet [25]. However, ICT has allowed diversification in the modalities and kinds of interventions available to support this population in the management of the demands inherent to their health condition. According to two literature reviews [26, 27], ICT-supported interventions aimed at PLHIV have shown promising results. However, until now their deployments and utilisations have been focused on adherence to HIV treatments.

Given the need to intervene actively on the modifiable risk factors in order to prevent the emergence and progression of comorbid problems among PLHIV and the advances in health behavior change interventions using ICT, we have developed a Web-based tailored intervention called *TAVIE en santé*. Its aims to support PLHIV in the adoption of health promoting behaviors such as ceasing smoking, engaging in physical activity, and following healthy eating habits.

### Prior work: TAVIE™ Web platform

In our previous work, we developed a virtual intervention concept called TAVIE (*Your life*) (French acronym for *Traitement Assistance Virtuelle Infirmière et Enseignement* or Treatment Virtual Nurse Assistance and Teaching) along with an innovative Web platform. Based on theories of behaviour change, TAVIE™ targets the development and consolidation of skills in order to enhance an individual’s ability to act. The aim is to provide individuals with real-time tailored support in managing the challenges inherent to their health conditions. The Web-based interventions developed on the TAVIE™ platform are unique in their tailored design that adapts content to users’ profiles and needs, the scientific rigour of the content and flexibility of use.

### Theoretical framework of *TAVIE en santé*

The theory of planned behavior (TPB) by Ajzen [28] has been used to predict and explain many health behaviors [29]. Based on the TPB, the adoption of a behavior is determined principally by the participant’s intention; however when adopting the behavior is not completely under volitional control, it is necessary to consider perceived behavior control. Perceived behavioral control, attitudes and subjective norms also play a role in behavior adoption, primarily through their effect on intention. External variables such as individual and environmental characteristics also influence these determinants. TPB as a guide in the development of the *TAVIE en santé* intervention, whose aim is to support PLHIV in quitting smoking, to practice physical activity and eating healthy foods. The following determinants of TPB are targeted to change participants’ behaviours: intention, attitude and perceived behavioural control.

### Study objective and hypotheses

This study aims to evaluate the effectiveness of a Web-based tailored intervention, *TAVIE en santé*, in supporting PLHIV to adopt health promoting behaviours, such as smoking cessation, being physically active and eating healthy.

The primary hypothesis is that at the 6-month follow-up, a significantly higher proportion of experimental group (EG) participants will have succeeded in changing the specific health behaviour that they individually selected (smoking cessation being physically active or healthy eating) when compared with the control group (CG) participants. The secondary hypothesis is that at the 3-month follow-up, compared with CG participants, EG participants will show significantly higher scores on intention, attitude, and perceived control regarding the adoption of their chosen health behaviour.

## Methods/Design

### Study design

An online randomized, controlled parallel-group trial will be conducted to evaluate the effectiveness of the Web-based tailored intervention *TAVIE en santé*. A convenience sample of participants will be randomly assigned either to an experimental group (*TAVIE en santé*) or to a control group (list of various Websites). Randomization will be performed as block randomization with a 1:1 allocation. Health behaviours, such as smoking cessation, being physically active and healthy eating are the primary outcomes. Cognitions (intention, attitude, and perceived behavioural control) towards the behaviour constitute the secondary outcomes.

### Population, study setting and sample size

The target population for this study is composed of Canadian PLHIV. According to the Public Health Agency of Canada (PHAC) [30], there were approximately 78,500 PLHIV living in Canada in 2013. Ontario accounted for the highest proportion of new reported PLHIV (39.6 % of reported positive HIV tests), followed by Quebec (21.7 %), and British Columbia (13.0 %). Thus, recruitment for this study will take place mainly in medical clinics and community organizations working with PLHIV in Ontario, Quebec, and British Columbia.

To participate, PLHIV must: 1) be 18 years of age or older; 2) be able to read and understand French or English; and 3) have access to the Internet.

To achieve the study’s objective, 375 participants per group are needed, for a total sample of 750 participants. This sample size was estimated to ensure 80 % statistical power for a 2-tailed chi-square test, at a significance level of α = 0.05, to detect any difference equal to or higher than about 10 % between the failure rates (failure to change the chosen behaviour-binary outcome) estimated in ITT analyses for the two randomization groups. This calculation takes into account the two likely limitations of the study: possible misclassification of self-reported outcomes and attrition. Similar to other online trials (Schubart review’s [31]: 15 trials reported less than 20 % of attrition), we expect up to 20 % attrition during the 6 months of follow-up.

### Interventions

During an individualized targeted online assessment, participants will have to choose a health behavior they wish to adopt (smoking cessation or increasing physical activity or adopting healthy eating). This assessment will also determine the participant’s level of intention, perceived behavioural control, and attitude towards to the adoption of the chosen behaviour.

#### Experimental group (EG): TAVIE en santé

*TAVIE en santé* is a Web-based tailored tri-component intervention addressing smoking cessation (SC), physical activity (PA) and healthy eating (HE, e.g. with a focus on making good choices regarding fat). Each component (SC, PA, HE) consists of seven interactive computer sessions lasting 5–10 min (total duration ≈ 50 min per component) hosted by a virtual nurse. The first and second sessions help the participant in identifying the benefits and drawbacks to adopting the chosen behaviour and in focusing on the benefits. The third and fourth sessions aim for the participant to identify barriers to adoption and ways to overcome them. In the fifth and sixth sessions, the participant formulates an action plan to adopt the behaviour. The seventh session acts as a booster.

Based on the online assessment of intention, perceived behavioural control, and attitude, three profiles are generated by a computer algorithm. The number of sessions, theory-based intervention methods and tailored message content will vary according to the profile. Profile 1 corresponds to participant with a low attitude toward changing the selected behavior, thus requiring a higher number of sessions (7 sessions). Profile 2 targets participants who have a low perceived behavioural control, which also requires a higher number of sessions (5 sessions). Profile 3 is for participants who have a high intention to modify their behaviour requiring a total of three sessions. Each profile receives specific, theory-based intervention methods and tailored messages. For example, belief selection and persuasive communication are used to change the participant’s attitude towards the behaviours; coping, planning and role modeling are used to improve perceived behavioural control and goal setting and implementation of intentions are used to act upon intentions.

During each session, the virtual nurse provides positive reinforcement, and feedback on the significant elements of the previous session. This ensures follow-up prior to each new session by verifying the participant’s understanding of the skills proposed and their application in daily life. Aside from delivering tailored teaching, the virtual nurse also refers to the experiences of other people who have been able to cope with similar situations successfully.

Access to the *TAVIE en santé* sessions will be unlimited in terms of intensity, frequency, and length of use for the duration of the study. After having completed their sessions, participants will have access to all previous sessions using a table of contents. *TAVIE en santé* is available both in French and in English. Nearly 350 Web pages have been created that contain 296 videos, 164 animated narrations and 84 PDF files containing information and tools. The Appendices 1 and 2 illustrate, respectively, a screenshot of a *TAVIE en santé* intervention Web page and examples of PDF files available to participants.

#### Control group (CG): list of predetermined Websites

The participants in the control group will be invited to visit five predetermined conventional health-related Websites offering libraries of information about the behaviour that they chose: smoking cessation, being physically active or healthy eating. These Websites were created by recognized Canadian agencies and associations (e.g.: Government of Canada, Canadian Cancer Society, Lung Association, Dieticians of Canada, Heart & Stroke Foundation, ParticipACTION). Therefore, their contents are reliable and the informations presented are of high quality. The lists of the Websites were validated by experts in each domain, including a nutritionist, kinesiologist, and smoking cessation nurse specialists.

There are two main differences between the control group and the experimental group: tailoring and the messenger. *TAVIE en santé* is a tailored intervention hosted by a virtual nurse that follows a decision tree, whereas the predetermined Websites offer general information not transmitted by a messenger.

### Measures/outcomes

All outcomes will be measured with a self-administered online questionnaire hosted on a secured Web page and will be collected at three times: baseline (t_0,_ 46 questions), and three (t_1,_ 26 questions) and 6 months (t_2,_ 34 questions) after baseline. Based on Prochaska’s [32] work on smoking cessation, physical activity and healthy eating, 6 months is the amount of time needed for this sort of health behaviour change to occur.

#### Primary outcome measures: health behaviours

The primary outcomes are the health behaviours (smoking cessation, physical activity, healthy eating). The sample will be divided into three subgroups by their choice of health behaviour. At 6 months post-baseline, the individual results for the chosen behaviours will be pooled and analyzed as a composite binary outcome (0 = failure to change the selected behavior, 1 = successful change of the selected behavior).

*Smoking cessation* will be measured with a single question: “In the past 7 days, have you had a smoke or even just a single puff?” Compared to biochemical verification (urinary nicotine test), this self reported-measure has a sensitivity of 96.9 % and a specificity of 93.4 % [33] and it correlates well with biochemical validation [34].

The level of *physical activity* will be measured through the adaptation of a procedure [35] elaborated and validated by Godin and al. [36, 37]. This measure has proven to be relevant in the context of participation in physical activity [38]. Participants will indicate, during a typical week and for each level of intensity (high, moderate, low), the frequency and the mean duration of physical activity practice.

Diet quality will be assessed via the short questionnaire *Starting the Conversation*. This is an 8-item simplified food frequency instrument [39] derived from a validated 54-item instrument [40]. Four items assessed the frequency of fat-related foods (e.g. fast food, chips, dessert, butter or meat fat), two items relate on vegetable/fruits, one item on sweetened beverages and one on protein (e.g. beans, chicken, fish).

#### Secondary outcomes measures: cognitions

*Intention*, *perceived behavioural control*, and *attitude* regarding the adoption of the chosen health behaviours will be measured, respectively, through 3, 3, and 5 items respectively on a 5-point bipolar scale, as recommended by Ajzen and Fishbein [41, 42]. All items reports to the future (“over the next 3 months”) and to the chosen health behaviour (a: stop smoking, b: do more physical activity, c: make better choices regarding fat in my diet). *Intention* is assessed by three items: “I intend to (a, b or c)”; 2) “I will (a, b or c)” and 3) “the chances that I will (a, b or c) are…”. *Perceived behavioural control* is assessed by three items: 1) “I feel that I will be able to (a, b or c)”; 2) “I am confident that I will succeed in (a, b or c)”; 3) “How much control do you have over (a, b or c)?” *Attitude* is measured by asking participants to respond–using five 5-point adjective pairs (unpleaseant/pleasant, useless/useful, bad/good, unsatisfying/satisfying, boring/interesting)–to the statement linked to their chosen behaviour “Smoking cessation would be for me…” or “Doing more physical activity would be for me…” or “Making better choices regarding fat in my diet would be for me…”.

#### Other measures

Sociodemographic data, such as sex, age, level of education, employment, and living situations will be assessed at baseline. Also, health indicators will be assessed through the self-report of HIV therapeutic regime and includes: HIV diagnosis, HIV viral load, CD4 count, blood pressure, blood cholesterol, diabetes, height/weight and general health condition perception.

### Recruitment

The study will be advertised in the three provinces where most of the target population lives: Ontario, Quebec and British Columbia. Recruitment will be mainly done offline in medical clinics, community agencies and community organizations working with PLHIV: health professionals and stakeholders will be invited to inform their clientele about this study using traditional methods, such as posters and information brochures. The project will also be adversited on the Websites of resources available to PLHIV (e.g. CATIE) where a hyperlink will be inserted to redirect PLHIV interested in participating to the study’s Website.

### Online data collection and allocation process

**Fig. 1:**
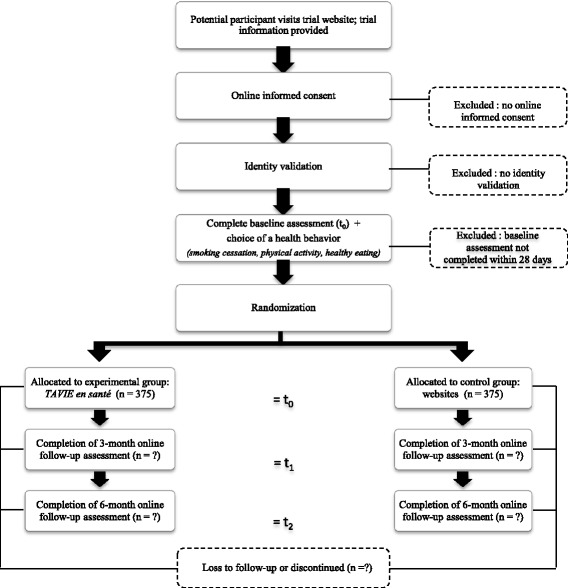
Schedule of enrolment, interventions, and assessments

The study will be conducted entirely online, the data collection process is very similar to the one described by Côté et al. 2012 [43]. The participant timeline, based on SPIRIT 2013 statement [44], is illustrated in Fig. 1.

Interested partcipants will be invited to visit the Website, which contains video clips about the study (see Appendix 3). After accepting the conditions and consenting, they will enroll in the study by providing an email address and a pseudonym. Each participant will be validated through an email address check [43]. After their enrolment via the study’s Website, participants will receive a hyperlink via email to invite them to complete a baseline questionnaire (t_0_) and to choose the behaviour that they wish to change. Only after the questionnaire has been completed will the participants be randomly assigned by the computer system to the EG or the CG. Participants will then receive an email containing the URL directing them to their intervention. Three (t_1_) and 6 months (t_2_) after the initial measurement, participants will complete the online questionnaires again.

Personalized reminder e-mails will automatically be sent at 14-day intervals prior to the measurement to encourage participants to perservere with the study. Other personalized reminder e-mails will be sent out to optimize participation in the interventions. Participants will be compensated for the time spent on the study with an Amazon.ca gift certificate of $20 after the second and third measurement times.

#### Randomization

As recommended in the CONSORT-EHEALTH statement, participants will be randomized only after they have completed the baseline assessment [45]. They will be randomly assigned to either control or experimental group with equal probabilities (1:1 allocation ratio). The allocation sequence has been generated by a permuted block randomization list. This method will ensure close balance of the number of participants in each group at any time during the study.

The allocation process (concealment and implementation) will be entirely computerized. The sequence will be programmed into the database intervention and the participants will be informed automatically by email about the group to which they are assigned.

#### Blinding

Given the differences between the two interventions arms described in the consent form, participants will be aware of the intervention they have been randomized into. However, it may be less evident to participants which group is the experimental group and which is the control group. In order to send the appropriate reminder emails, the research coordinator will be aware of the participant’s assignment. During data entry and analysis, the research team will be blinded to participant’s group assignement (one database will contain participants’ information and another only the collected datas).

### Analysis

Descriptive statistics will be used to compare the participants in the two groups with means, medians, standard deviations, and inter-quartile ranges for continuous and frequency distributions for categorical and binary variables. Any variable for which the difference between the two arms is considered clinically important will be adjusted through multivariable analyses. The principal analyses will focus on testing the hypothesized differences between the two trial groups using the Intention-to-Treat (ITT) analysis.

The primary outcome of the success/failure in the adoption of the chosen health behaviour at 6 months will be analyzed as a binary outcome. Accordingly, the primary hypothesis will be tested with the 2-tailed chi-square test, with 1 degree-of-freedom (df), at α = 0.05 significance level. Next, to adjust the intervention effect for potential confounders, we will build a multivariable logistic regression with adjustment for any baseline characteristics for which the descriptive analyses indicate a clinically important difference between the two groups. The multivariable model will also adjust for baseline values of the chosen health behaviour. In the multivariable logistic regression, the effect of the intervention will be estimated by the adjusted Odds Ratio, with 95 % CI, and its statistical significance will be tested by the model-based 1-df Wald chi-square test at 2-tailed α = 0.05. During ITT analyses, all participants will be assessed in their respective randomization group as to whether they are following the procedure or have been lost to attrition during the follow-up. In this manner, all participants lost to follow-up before 6 months will be assigned a ‘failure’, regardless of the group to which they were assigned randomly. This approach is conservative because it reduces the estimated difference between the two randomization groups.

The secondary outcomes of intention, perceived behavioural control, and attitude, will be evaluated at the 3-months follow-up, and analysed as continuous variables. Thus, the same analytical methods will be used for all these outcomes. The distribution of the outcome scores will be assessed for normality of residuals using the Shapiro-Wilk test. We will assign the individual baseline score for a given outcome to participants who fail to provide data for a given outcome. To test the statistical significance of the un-adjusted difference between the mean values of a given (possibly transformed) outcome observed in the two trial arms at 3 months, we will use the 2-tailed independent-groups student’s *t*-test at α = 0.01. Multivariable linear regression will be used to adjust the estimation and testing of the intervention effect for potential confounders, for which the descriptive analyses indicate a clinically important difference between the two groups. In multivariable linear regression, the effect of the intervention will be estimated by the adjusted mean difference, with 95 % CI, and its statistical significance will be tested by the model-based F-test with 1-df in the numerator, at 2-tailed (Bonferoni-corrected) α = 0.01.

### Ethics

#### Research ethics approval

In Quebec, this research protocol has been approved by the Research Ethics Board (REB) of the Centre Hospitalier de l’Université de Montréal (CHUM) following a multicentre research project protocol. The CHUM REB’s approval will then be forwarded to the other local study sites in Ontario and British Columbia, which will then seek the approval of their respective boards.

#### Consent

Due to the online nature of this study, the informed consent will be obtained online. To pique the interest of potential participants and render the study’s Website more dynamic and interactive, video clips will be posted, containing information about the study. In one of them, a peer will present specific information about the study (goal, modalities of participation, eligibility criteria, confidentiality, financing, etc.). Consent to participate will be presented in the form of a statement to be read and approved by clicking on a button “I agree to participante” [43]. A “Withdraw” page will always be available so that participants have the freedom to withdraw from the study at any time.

#### Confidentiality

The data collected online will be stored on a secure server located at the Research Centre of the CHUM and accessible to the research coordinator and principal investigator only. To protect the participants’ identity, an e-mail address and a login name known only to the research coordinator and the system administrator will be requested of participants. This information is necessary to ensure follow-up while respecting confidentiality, as pointed out by Michalak and Szabo [46]. Firewalls, data encryption, password protection, and separation of socio-demographic data from experimental data will reduce the risk of unauthorized access to confidential content by a third party [47, 48].

## Results

### Study Website description

A Website was created to promote and conduct this online study (see Appendix 3). As suggested by Hershberger and Coll. (2011), this Website was designed to communicate information, establish credibility, and facilitate contact [49]. Videos, animation, text, and PDF files are used to describe the purpose and implications of the study to the participants. All materials were prepared with ease of comprehension in mind. To inform participants about the partners of the study, we created a page to display the logos of the University’s affiliations of the investigators, financing organizations and recruiting sites. To facilitate communication, a “Contact Us” navigation tab, containing information on technical support or the reaching the research coordinator, is visible in every page of the Website.

### Study flow diagram

**Fig. 2:**
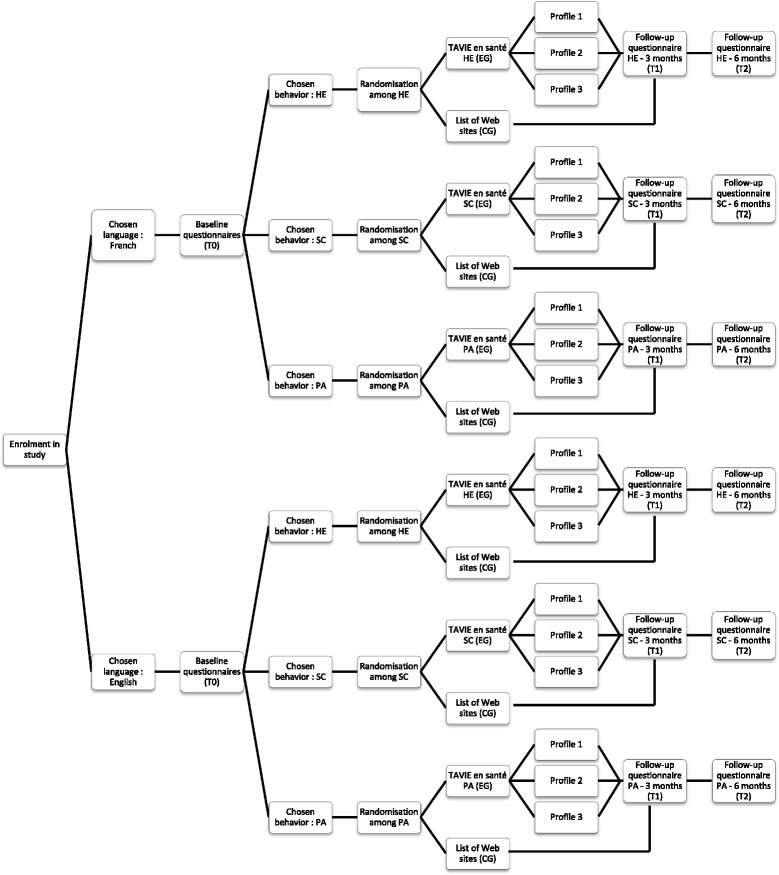
Study flow diagram

Once participants enrol in the study, they will follow different “pathways” over the 6 months of the study. The pathways depend on the chosen language and behaviour. During the enrolment process, participants will choose the language they prefer (French or English). The behaviour they choose in the baseline questionnaire (smoking cessation (SC), being physically active (PA), following a healthy eating (HE)) will impact randomization and follow-up questionnaires (t_1_ and t_2_). Therefore, there will be six pathways, as illustrated in Fig. 2.

## Discussion

### Challenges and sources of bias

The study will be conducted entirely online, which poses various challenges in terms of participants’ selection and data collection [50]. One of our earlier online studies established the feasibility of the proposed research methodology and will serve as a guide to participants’ selection and data collection [43]. For instance, to prevent participants from registering more than once, the computer system will screen them to verify whether they meet the eligibility criteria. The design of the online questionnaire will only allow answering one question at a time and will not allow backtracking.

The study design will be controlled as best as possible but participants will not be totally blinded to group assignment. The CG will receive a detailed list of Websites providing relevant information related to health behaviour and available resources and services. Group equivalence will be ensured through random assignment and–if necessary–statistical adjustments, through multivariable regression modeling, for any potential imbalance in the distribution of any relevant baseline characteristic will be performed. Measures will be taken to ensure intervention fidelity/consistency by documenting all aspects of the intervention. Moreover, the intervention’s parameters will be recorded (number of pages visited), thus making it possible to obtain a reliable picture of the actual intervention intensity: duration and frequency of the intervention.

To reduce contamination bias, participants will be sensitized to the importance, from a research viewpoint, of not sharing with others the intervention material to which they will have access during the study. In any event, given that one of the particularities of the intervention is that it is “tailored”, the personalized feedback and information received is unlikely to be relevant to another participant. A selection bias will be inevitable because participants invited to participate in the study must necessarily have Internet access [51]. Given that the size and demographic characteristics of the online population are different from those of the off-line population [52–54], the results of the study will not be generalizable to PLHIV as a whole [55]. Given that many PLHIV are living on fixed incomes and for whom personal internet access may not be available, this constitutes a limitation of the planned intervention; however, for those who are able and willing to use technology, *TAVIE en santé* provides a potential health promoting and reinforcing resource.

### Projected impacts, benefits, and transfer of the study

Until now, most of the studies testing the efficacy of Web-based tailored health behaviour change interventions have been designed and conducted among the general population, were more preventive in nature and did not involve support for complex tasks required in self-management of chronic conditions (Lustria et al., 2013). According to Murray (2012), Web-based interventions for people with long-term conditions remain a challenge and may be less successful. The aim of the study is to evaluate an innovative Web-based tailored intervention for encouraging PLHIV to adopt specific health behaviours. This study will yield new results about the efficacy of Web-based tailored health behaviours change interventions in the context of chronic diseases (chronically ill patients).

Given the current short supply of specialists in healthcare and in the area of HIV-related services, *TAVIE en santé* offers clinicians an complementary instrument to support their professional practice and meet their clientele’s needs with a minimum investment. As it consists essentially of simulated interactions between a virtual nurse and the PLHIV, all that is needed is to provide minimal remote computer support services available in case of technical problems. This computer innovation affords the system administrator the flexibility to add and modify page without always having to resort to a Web developer. Thus, it reduces the costs of the system and facilitates the implementation of solutions over the long term in a normal context of clinical practice.

## Appendix 1

*TAVIE en santé* screenshot.

## Appendix 2

Examples of *TAVIE en santé* PDF tool for participants.

## Appendix 3

Study website screenshot.

## Abbreviations

ART: Antiretroviral therapy
CI: Confidence interval
CHUM: Centre Hospitalier de l’Université de Montréal
HE: Healthy eating
ICT: Information and communications technologies
ITT: Intention-to-Treat
PA: Physical activity
PLHIV: Persons living with HIV
REB: Research Ethics Board
SC: Smoking cessation
TPB: Theory of planned behavior
